# Identification of Neotropical *Culex* Mosquitoes by MALDI-TOF MS Profiling

**DOI:** 10.3390/tropicalmed8030168

**Published:** 2023-03-13

**Authors:** Monique Melo Costa, Amandine Guidez, Sébastien Briolant, Stanislas Talaga, Jean Issaly, Halima Naroua, Romuald Carinci, Pascal Gaborit, Anne Lavergne, Isabelle Dusfour, Jean-Bernard Duchemin, Lionel Almeras

**Affiliations:** 1 Unité de Parasitologie et Entomologie, Département de Microbiologie et Maladies Infectieuses, Institut de Recherche Biomédicale des Armées, 13005 Marseille, France; 2 Aix Marseille Univ, IRD, SSA, AP-HM, VITROME, 13005 Marseille, France; 3 IHU Méditerranée Infection, 13005 Marseille, France; 4 Unité d’Entomologie Médicale, Institut Pasteur de la Guyane, 97354 Cayenne, France; 5 Unité de Paludologie et d’Entomologie Médicale, CERMES, YN034, Niamey BP 10887, Niger; 6 Laboratoire des Interactions Virus-Hôtes, Institut Pasteur de la Guyane, 97354 Cayenne, France

**Keywords:** *Culex* species, neotropical mosquito, French Guiana, MALDI-TOF MS, proteomic tool, arbovirus, monitoring

## Abstract

The mosquito (Diptera: Culicidae) fauna of French Guiana encompasses 242 species, of which nearly half of them belong to the genus *Culex*. Whereas several species of *Culex* are important vectors of arboviruses, only a limited number of studies focus on them due to the difficulties to morphologically identify field-caught females. Matrix-assisted laser desorption/ionization time-of-flight mass spectrometry (MALDI-TOF MS) has been reported as a promising method for the identification of mosquitoes. *Culex* females collected in French Guiana were morphologically identified and dissected. Abdomens were used for molecular identification using the *COI* (cytochrome oxidase 1) gene. Legs and thorax of 169 specimens belonging to 13 *Culex* species, (i.e., *Cx. declarator*, *Cx. nigripalpus*, *Cx. quinquefasciatus*, *Cx. usquatus*, *Cx. adamesi*, *Cx. dunni*, *Cx. eastor*, *Cx. idottus*, *Cx. pedroi*, *Cx. phlogistus*, *Cx. portesi*, *Cx. rabanicolus* and *Cx. spissipes*) were then submitted to MALDI-TOF MS analysis. A high intra-species reproducibility and inter-species specificity of MS spectra for each mosquito body part tested were obtained. A corroboration of the specimen identification was revealed between MALDI-TOF MS, morphological and molecular results. MALDI-TOF MS protein profiling proves to be a suitable tool for identification of neotropical *Culex* species and will permit the enhancement of knowledge on this highly diverse genus.

## 1. Introduction

The neotropical region is inventoried as a hotspot of mosquito (Diptera: Culicidae) species richness. French Guiana (hereafter FG) is a department of France situated in South America that harbors one of the highest relative species densities of mosquitoes in the world [1,2]. Currently, 242 mosquito species, classified into 22 genera are known in this French territory [3]. Some species are medically important because they are proven vectors of human pathogens. For example, several *Anopheles* species are vectors of malaria pathogens in FG [4,5,6,7]. *Yellow Fever* (*YFV*), *dengue* (*DENV*), *chikungunya* (*CHIKV*) and *Zika* (*ZIKV*) viruses are transmitted by the day-biting *Aedes* (*Stegomyia*) *aegypti (Linnaeus, 1762)* in urban areas [8,9,10].

Containing 788 species classified into 26 subgenera worldwide, the genus *Culex* (*Cx*.) is one of the largest groups of the Culicidae family [11]. In FG, 104 species belong to the genera *Culex* [3], with a majority of species linked to two subgenera named *Culex* (*Cux*.) and *Melanoconion* (*Mel*.). In the *Culex* subgenus, many species are vectors of arboviruses and parasites. *Culex* (*Cux*.) *quinquefasciatus* (Say, 1823) was identified as a vector of the nematode *Wuchereria bancrofti* (Cobbold, 1887)*,* an agent of lymphatic filariasis in FG [12] and as a primary vector in northeast Brazil [13]. On a larger scale, this anthropophilic mosquito is also known to be a vector of Western equine encephalitis virus (WEEV, *Alphavirus*) and many viruses from the *Flavivirus genus,* such as Saint-Louis encephalitis *virus* (SLEV) and West Nile virus (WNV); additionally, it is also a vector of nematode *Dirofilaria immitis* (Railliet & Henry, 1911)*,* that could infect both humans and dogs [14,15,16,17]. In South America, *Cx.* (*Cux*.) *declarator* (Dyar & Knab, 1906) is a vector of SLEV and Bussuquara virus (BSQV, *Flavivirus* genus) [18]. The Cabassou virus (CABV, *Alphavirus* genus) was already isolated from *Culex* (*Cux*.) *nigripalpus* Theobald, 1901 in FG [19] and this virus is a recognized vector for members of *Flavivirus* genus with SLEV, WNV and ZIKV and for members of the *Alphavirus* genus with Venezuelan equine encephalitis virus (VEEV) and Eastern equine encephalitis virus (EEEV) in America [20,21]. *Culex* (*Cux*.) *usquatus* (Dyar, 1922) belonging to the Coronator Complex is not a recognized as vector, but the females are indistinguishable from *Cx.* (*Cux*.) *coronator* (Dyar & Knab, 1906) which is known as vector of WNV [22] and may participate in the transmission of SLEV, VEEV and Mucambo Virus (MUCV, *Alphavirus* genus) [23,24].

In the *Melanoconion* subgenus, *Cx*. (*Mel*.) *portesi* (Senevet & Abonnenc, 1941) and *Cx*. (*Mel*.) *spissipes* (Theobald, 1903) are highly suspected to be natural vectors of Tonate virus (TONV), which belongs to the *Alphavirus* genus in FG [25,26,27], but is also found to be infected by *Bunyavirus* (Caru, group Guama, Caraparu and group C) [19]. *Culex* (*Mel*.) *portesi* is a recognized natural vector for MUCV in Trinidad [28] and is a natural vector for the disease caused by El Huayo virus (a group C *Orthobunyavirus*) in Peru [29]. Ecological studies and the isolation of many arboviral species in *Cx*. (*Mel*.) *portesi* rank it as high potential invertebrate hosts of numerous viruses [19]. *Culex* (*Mel.*) *adamesi* (Sirivanakarn & Galindo, 1980), *Cx.* (*Mel.*) *dunni* (Dyar, 1918), and *Cx.* (*Mel.*) *pedroi* (Sirivanakarn & Belkin, 1980) are natural enzootic vectors of VEEV in Columbia, Peru and Panama [30,31,32]. *Culex* (*Mel*.) *dunni* was also found to be infected with Pacora virus (PCAV, *Bunyavirus*-like) in Panama [33]. A last example is *Cx.* (*Mel*.) *idottus* (Dyar, 1920), which is a caiman-biting mosquito involved in the transmission of *Hepatozoon caimani* (Carini, 1909) and is suggested as a potential vector of WNV [34].

With global warming, deforestation and urbanization, the distribution areas of mosquitoes change, colonizing new territories. Such changes could promote the emergence of new mosquito vectors due to their exposure to disease agents and hosts, inducing potential outbreaks. To manage and to prevent epidemic emergence, the monitoring and accurate identification of *Culex* mosquitoes at the species level remains essential. Presently, morphological and molecular methods are the main strategies available for the identification and classification of *Culex* species.

Morphological identification at the species level of *Culex* mosquitoes requires the careful dissection and mounting of male genitalia by an entomological expert because of the presence of slight morphologic characters that allow the identification of sibling species of the genera [35]. Female identification is even harder because morphological characters may be either polymorphic or isomorphic among distinct species [36]. This approach is labor-intensive and time-consuming and, therefore, might not be adapted for routine identification of *Culex* females. Furthermore, field-caught specimens are rarely in perfect condition, which precludes any reliable morphological identification. For damaged mosquitoes or for immature stages, molecular tools can be an interesting alternative, especially to distinguish morphologically close species [37]. Barcoding and metabarcoding markers which combine DNA barcoding with high-throughput sequencing are sometimes used for delimitation and identification of species. However, species-level identification depends heavily on the choice of marker and the selected primer pair, often with a trade-off between successful species amplification and taxonomic resolution. Variation of a partial sequence of the cytochrome *c* oxidase subunit I (*COI*) gene is often used for the identification of mosquito species and contributes to discovering cryptic diversity [38]. If on the one side, the *COI* barcode can be successfully used for delimiting and identifying numerous mosquito species, on the other hand, recent studies on the *Culex* subgenus identification with this fragment indicate a poor resolution in separating species among complexes [36]. To improve species identification, multiple marker/primer pairs are often recommended and this remains an expensive method for screening large samples [39].

In the last decade, an innovative and cheaper proteomic tool named MALDI-TOF MS profiling emerged for rapid mosquito species identification [40]. The principle is based on the matching of species-specific protein signatures of a specimen with a reference spectra database. This promising tool has been reported to distinguish cryptic species of the *Anopheles* genus with high efficiency [41]. Already used successfully for different taxonomic groups of insects, including culicids [42] and phlebotomids [43], standardization of protocols and optimized procedures could enable sharing all MS spectra references and lead to the creation of an international MS database [44].

Thus, the aim of the present study was to assess whether this proteomic tool could distinguish species from the neotropical *Culex* genus. In this way, field-caught mosquitoes from FG of the *Culex* genus were selected, classified by morphological criteria and confirmed by molecular barcoding. The legs and thoraxes of the selected *Culex* specimens were then submitted independently to MALDI-TOF MS to assess intra-species reproducibility and inter-species specificity of MS spectra. The establishment of such an innovative tool for *Culex* mosquito identification should improve studies on this genus which are often hampered by the complexity of current methods for accurate classification.

## 2. Materials and Methods

### 2.1. Mosquito Collection

Adult mosquitoes were collected from different sites of the coastal floodplains of FG. These mosquito collections occurred during entomological surveys conducted from November 2018 to January 2020, using backpack aspirators and CDC light traps baited (or not) with dry ice. Female specimens of *Culex* were sorted out and morphologically identified (whenever possible) using taxonomic keys [45,46,47,48,49] under a Leica M165 C stereomicroscope at a magnification up to ×120 (Leica Microsystems, Wetzlar, Germany). Mosquitoes were identified to the species level when possible, otherwise to the genus level followed by the suffix ‘sp.’ combined with a number. The abdomen, thorax and legs of each mosquito specimen were dissected and kept individually at −20 °C for subsequent molecular and MALDI-TOF MS analyses. A total of 169 selected females were used in this study. The collection sites are located in urban and peri-urban coastal areas where regular monitoring of Culex species is performed in order to estimate the risk of arboviruses transmission (e.g., Tonate virus).

### 2.2. Molecular Identification of Mosquitoes

DNA was individually extracted from the abdomen of each mosquito specimen using the QIAamp DNA tissue extraction kit (Qiagen, Hilden, Germany) following the manufacturer’s instructions. Sequences of a fragment of the cytochrome c oxidase subunit I (*COI*) gene from extracted DNA of each mosquito were amplified using DNA-barcode primers forward LCO1490 (5′-GGT CAA CAA ATC ATA AAG ATA TTG G-3′) and reverse HC02198 (5′-TAA ACT TCA GGG TGA CCA AAA AAT CA-3′), according to protocols previously described [50]. After the expected amplified DNA fragment size (658 pb) was verified by electrophoresis (1.5% agarose gel), samples were sent for sequencing. Sequences were edited using the Molecular Evolutionary Genetics Analysis 11 (MEGA) X64 software, assembled and aligned in Clustal ω2 algorithm using the default parameters.

A reference database of *Culex COI* sequences obtained from sequences accessible in the Barcode of Life Data Systems (BOLD) [51] and in *GenBank*^®^ [52] was used for identification and to construct a Maximum Likelihood tree using a General Time Reversible (GTR) model +G+I (Gamma distribution; evolutionarily Invariable). Support for internal nodes was estimated using the nonparametric bootstrap method with 1000 replicates. By using the Nucleotide Basic Local Alignment Search Tool (BLAST) [53], the identity percentage and query coverage parameter were also extracted for each sample. Only specimens with identity >97% were selected for this study. Each specimen was then assigned to a mosquito species name. Species identification through DNA barcoding was used for all specimens including undetermined *Culex* female specimens but also for confirmation of specimens already identified by morphology.

### 2.3. Phylogenetic Tree Analyses Based on COI Sequences for MALDI-TOF Comparison

*COI* sequences from specimens included in the MALDI-TOF reference database were used for the creation of phylogenetic trees. Sequences were aligned through the Clustal ω2 algorithm in MEGA X and applied to construct a Maximum Likelihood tree for thoraxes with similar analyses used for identification of the *COI* sequences to species level.

### 2.4. Sample Homogenization and MALDI-TOF MS Analysis

The thorax and legs of each mosquito specimen were prepared for MALDI-TOF MS as previously described [54,55]. After homogenization of each sample with mix buffer (1:1 solution of 70% (*v*/*v*) formic acid and 50% (*v*/*v*) acetonitrile), 1 µL of the supernatant was spotted on the MALDI-TOF steel target plate (Bruker Daltonics, Wissembourg, France) in quadruplicate and air-dried. The spots were covered with 1 µL of matrix solution containing saturated α-cyano-4-hydroxycinnamic acid, accordingly to previous work [56]. To control matrix quality (i.e., absence of MS peaks due to matrix buffer impurities) and MALDI-TOF apparatus performance, the matrix solution was loaded in duplicate onto each MALDI-TOF plate alone. Likewise, the thorax and legs from one fresh specimen of *Ae.* (*Stg.*) *aegypti* Bora-Bora strain were loaded on each plate as a quality control of sample preparation and MS spectra acquisition.

### 2.5. MALDI-TOF MS Parameters

Protein mass profiles were obtained using a MicroFlex LT MALDI-TOF Mass Spectrometer (Bruker Daltonics, Germany), with detection in the linear positive-ion mode at a laser frequency of 50 Hz within a mass range of 2–20 kDa. The setting parameters of the MALDI-TOF MS apparatus were identical to those previously used [57]. MS spectra were acquired automatically using the AutoXecute of the Flex Control v.2.4 software (Bruker Daltonics).

### 2.6. MS Spectra Analysis

MS spectra profiles were controlled visually first with flexAnalysis v.3.3 software (Bruker Daltonics), then exported to ClinProTools v.2.2 and MALDI-Biotyper v.3.0 software (Bruker Daltonics) for data processing (smoothing, baseline subtraction, peak picking). MS spectra reproducibility was assessed by the comparison of the average spectral profiles (MSP, Main Spectrum Profile) obtained from the four spots for each specimen according to body part with MALDI-Biotyper (Bruker Daltonics). Additionally, mosquito body part MS spectra reproducibility and specificity were assessed using cluster analyses (MSP dendrogram) and the composite correlation index (CCI) tool. The MSP dendrograms were performed from clusters of the specimens, based on comparison of their protein mass profile (i.e., their mass signals and intensities). The CCI tool from MALDI-Biotyper was also used to assess the spectral variations within and between each sample group, as previously described [58]. Higher correlation values (expressed as the mean ± standard deviation, SD) reflecting higher reproducibility for the MS spectra, were used to estimate MS spectra distance between species for each body part. In addition, ClinProTools was used to identify discriminatory peaks among the 13 mosquito species for each body part. The top five of the most intense MS peaks per mosquito species and per body part were analyzed with ClinProTools to estimate their performance to discriminate the *Culex* species. The default parameter settings in ClinProTools for spectrum preparation were applied as previously described [54]. Based on the peak list obtained per species for each body part, the top five most intense m/z peaks were selected for inclusion in the genetic algorithm (GA) model. The peaks selected by the operator provided a recognition capability (RC) value bound to the highest cross-validation (CV) value. The presence or absence of all discriminating peak masses generated by the GA model was controlled by comparing the average spectra from each species per body part.

### 2.7. Database Creation and Blind Tests

The MS reference database (DB) was created using spectra from paired legs and thoraxes of one to four specimens per species using MALDI-Biotyper (Bruker Daltonics). MS spectra were created with an unbiased algorithm using information on the peak position, intensity and frequency. A total of 58 MSP from thoraxes or legs were included in the reference MS spectra DB (Table 1). The raw MS spectra from legs and thoraxes of *Culex* mosquitoes are provided for free use (Supplementary File S1). MS spectra from mosquito legs and thoraxes of the 111 remaining specimens were tested against this in-house MS reference spectra DB. The reliability of species identification was estimated using the log score values (LSVs) obtained from the MALDI Biotyper software v.3.0, which ranged from 0 to 3. According to previous studies [42,56,57], LSVs greater than 1.8 were considered reliable for species identification. Data were analyzed with Prism software v.7.00 (GraphPad, San Diego, CA, USA).

## 3. Results

### 3.1. Morphological and Molecular Identification of Culex Specimens

Field mosquito catching was performed from 2018 to 2020 in three distinct areas of FG (Table 1). Among the mosquitoes captured, only *Culex* female specimens were selected for the present study. The morphological identification was applied to 205 females. However, 30% (61/205) of the specimens were unsuccessfully classified to species level. Morphological identification to species level was established for five species (*Cx.* (*Cux.*) *quinquefasciatus*, *Cx.* (*Cux.*) *usquatus, Cx.* (*Mel.*) *pedroi* and *Cx.* (*Mel.*) *portesi*) and proved to be validated by molecular identification. All *Culex* specimens were then subjected to molecular analysis by DNA barcoding, using the *COI* gene marker, and each *COI* sequence was queried against the BOLD system. Reliable species identifications were obtained for 169 females with identity and coverage ranges of 97–100% and 100%, respectively (Table 1). *Culex* females belonged to 13 distinct species classified into two subgenera. Four species were categorized into the *Culex* subgenus (*Cx.* (*Cux.*) *declarator*, *Cx.* (*Cux.*) *nigripalpus*, *Cx.* (*Cux.*) *quinquefasciatus* and *Cx.* (*Cux.*) *usquatus*), and the nine remaining species were classified into the *Melanoconion* subgenus (*Cx.* (*Mel.*) *adamesi*, *Cx.* (*Mel.*) *dunni*, *Cx.* (*Mel.*) *eastor* (Dyar, 1920), *Cx.* (*Mel.*) *idottus*, *Cx.* (*Mel.*) *pedroi*, *Cx.* (*Mel.*) *phlogistus* (Dyar, 1920), *Cx.* (*Mel.*) *portesi*, *Cx.* (*Mel.*) *rabanicolus* (Floch & Abonnenc, 1946) and *Cx.* (*Mel.*) *spissipes*). According to our data set availability, 1 to 34 specimens per species were acquired. Thirty-four *Culex* specimens encompassing the 13 species and used for MALDI-TOF MS reference database were selected for the phylogenetic analysis based on *COI* gene sequences (Supplementary File S2). Specimens were successfully assembled and classified by species.

### 3.2. Reproducible and Specific MS Spectra from Two Culex Body Parts

**Figure 1:**
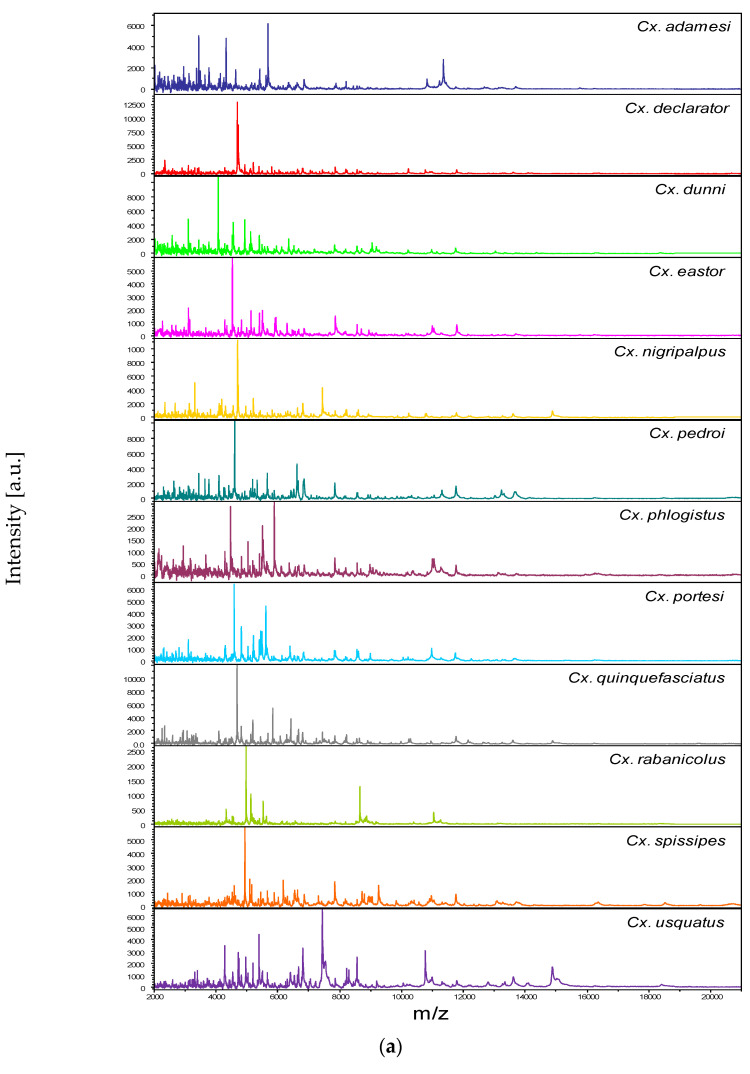

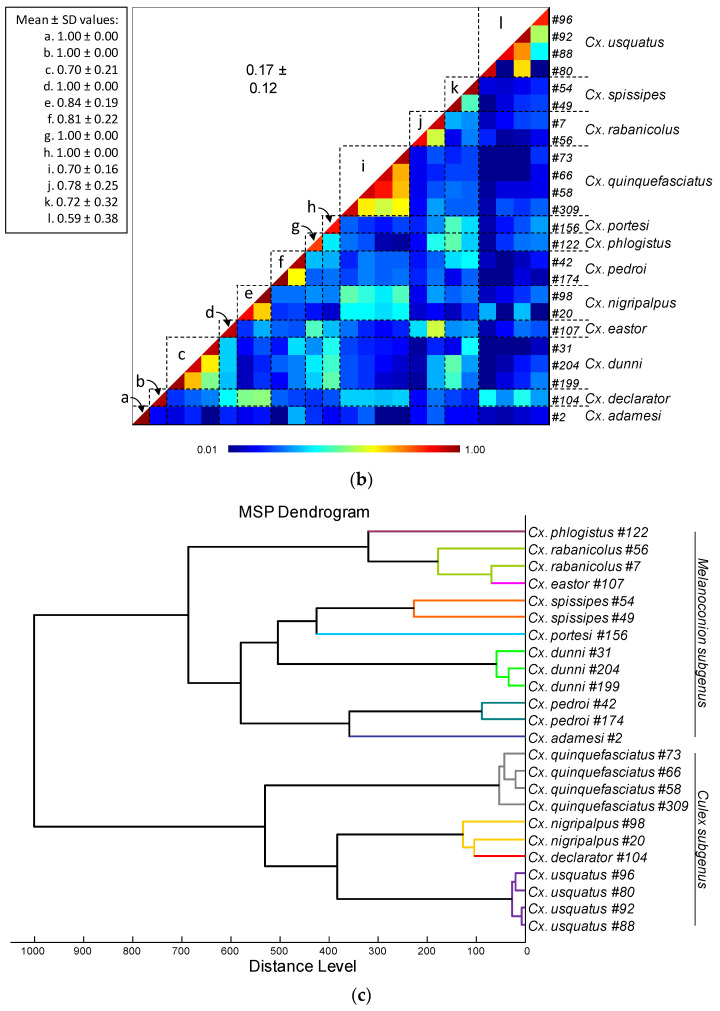
Comparison of MALDI-TOF MS spectra from legs of *Culex* mosquitoes. (**a**) Representative legs MS spectra of *Culex* species. The name of each species was indicated on the right corner of each spectrum. (**b**) Composite Correlation Index (CCI) matrix value representing the levels of legs MS spectra reproducibility among *Culex* specimens of the same species and between species. The 24 specimens from the 12 *Culex* species, which were selected as reference MS spectra introduced in the MS DB, are shown. The levels of MS spectra reproducibility are indicated in red and blue, revealing relatedness and incongruence between spectra, respectively. The values correspond to the mean coefficient correlation and respective standard deviations obtained for paired condition comparisons. CCI was calculated with MALDI-Biotyper v.3.0 software. (**c**) MSP dendrogram of MALDI-TOF MS spectra from legs of *Culex* mosquitoes from the 24 *Culex* specimens selected as reference MS spectra. The *Culex* and *Melanoconion* subgenera were indicated at the right. The distance units correspond to the relative similarity of MS spectra. The dendrogram was created by Biotyper v.3.0 software. The same color code was used for specimens of the same *Culex* species between panels (**a**,**c**). a.u., arbitrary units; *Cx.*, *Culex*; MSP, Main Spectrum Profile; m/z, mass-to-charge ratio; SD, standard deviation.

**Figure 2:**
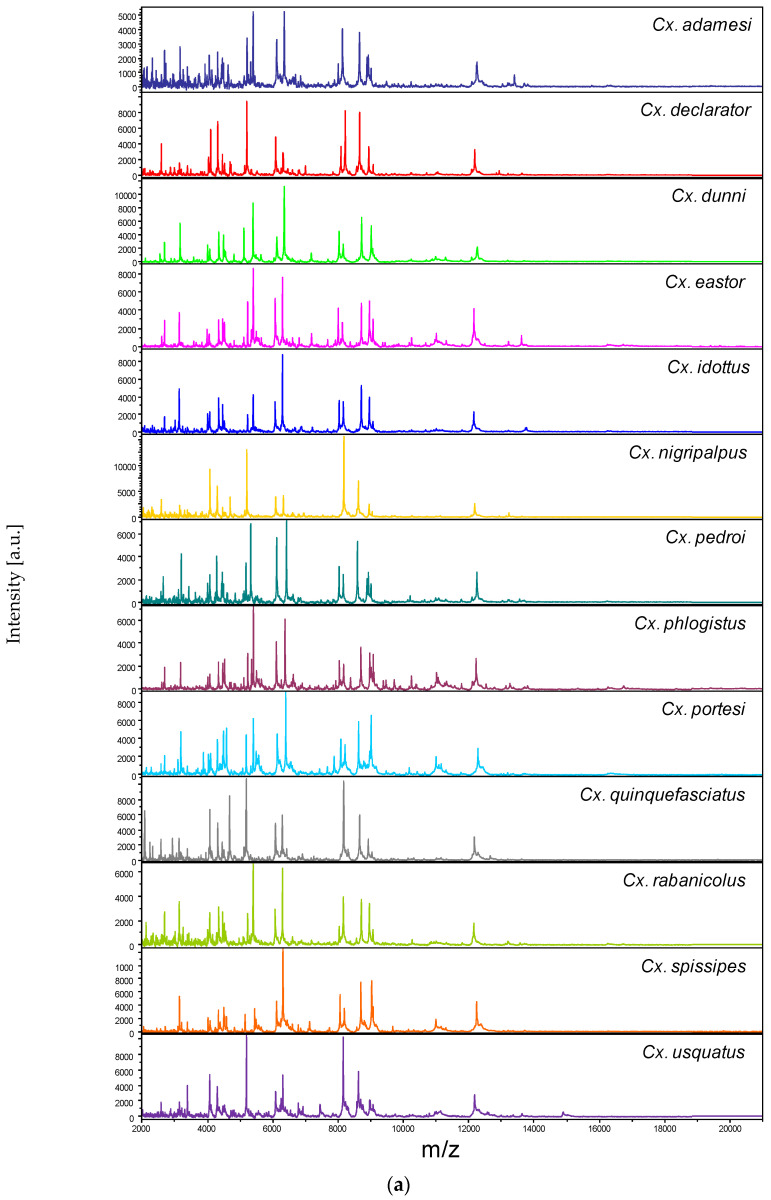

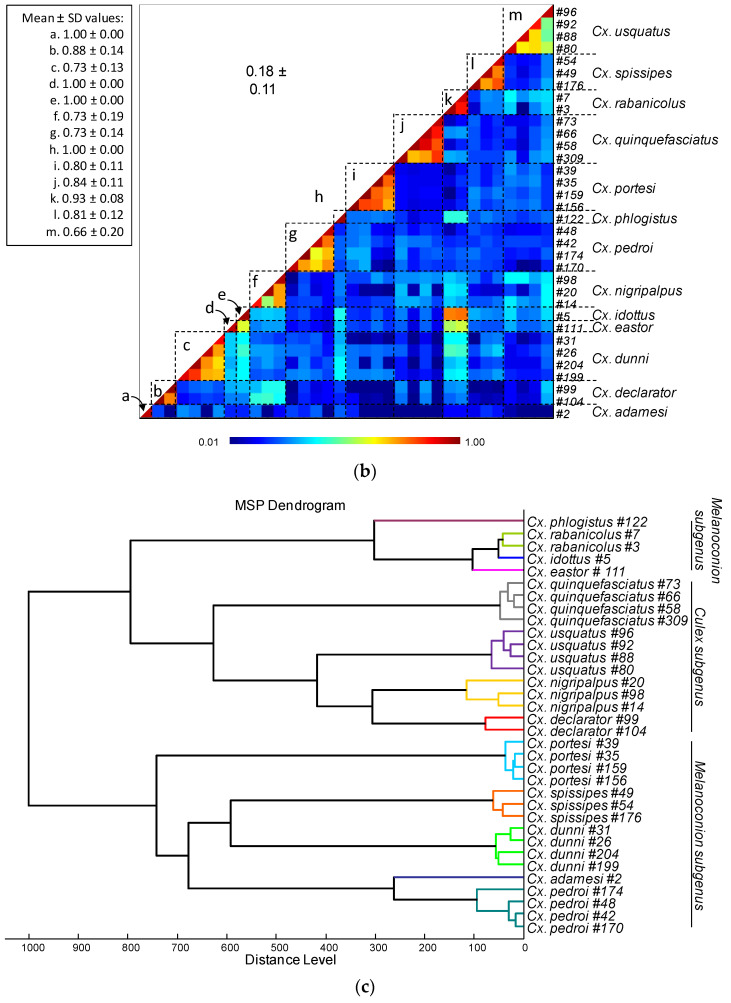
Comparison of MALDI-TOF MS spectra from thoraxes of *Culex* mosquitoes. (**a**) Representative thoraxes MS spectra of *Culex* species. The name of each species was indicated on the right corner of each spectrum. (**b**) Composite Correlation Index (CCI) matrix value representing the levels of thoraxes MS spectra reproducibility among *Culex* specimens of the same species and between species. The 34 specimens from the 13 *Culex* species, which were selected as reference MS spectra introduced in the MS DB, are shown. The levels of MS spectra reproducibility are indicated in red and blue, revealing relatedness and incongruence between spectra, respectively. The values correspond to the mean coefficient correlation and respective standard deviations obtained for paired condition comparisons. CCI was calculated with MALDI-Biotyper v.3.0 software. (**c**) MSP dendrogram of MALDI-TOF MS spectra from thoraxes of *Culex* mosquitoes, from the 34 *Culex* specimens selected as reference MS spectra. The *Culex* and *Melanoconion* subgenera were indicated at the right. The distance units correspond to the relative similarity of MS spectra. The dendrogram was created by MALDI-Biotyper v.3.0 software. The same color code was used for specimens of the same *Culex* species between panels (**a**,**c**). a.u., arbitrary units; *Cx.*, *Culex*; MSP, Main Spectrum Profile; m/z, mass-to-charge ratio; SD, standard deviation.

Among the 1352 MS spectra (169 specimens × two body parts × four replicates) acquired, MS profiles of high intensity (>3000 a.u.) were reached for the vast majority of the samples (>95%). The samples presenting MS spectra with low intensity were not selected for introduction as reference spectra in the MS DB. For example, MS spectra of legs from the two *Cx.* (*Mel.*) *idottus* specimens were excluded from the analysis due to the low intensity and inter-sample heterogeneity of MS profiles (Supplementary File S3). Then, in total, MS spectra of 12 and 13 *Culex* species were analyzed for legs and thoraxes, respectively. The visual comparison of MS profiles acquired for legs (Figure 1a) and thoraxes (Figure 2a) differed importantly among the *Culex* species. To assess the reproducibility and specificity of MS spectra per body part according to species, CCI and cluster analyses were carried out. MS spectra of high intensity (>3000 a.u.) from paired legs (*n* = 24) and thoraxes (*n* = 34) of one to four specimens per species were selected for these analyses. The mean CCI values obtained among MS spectra per body part and per species were elevated, ranging from 0.59 to 0.84 for legs (Figure 1b) and from 0.66 to 0.93 for thoraxes (Figure 2b). Conversely, the mean CCI values obtained between species for legs and thoraxes were very low (<0.20), supporting the high MS spectra species-specificity. Nevertheless, it is interesting to note that elevated CCI values were observed for thoraxes MS spectra between *Cx.* (*Mel.*) *idottus* and *Cx.* (*Mel.*) *rabanicolus* (mean ± SD: 0.77 ± 0.01).

The MSP dendrograms created per body part with the same MS spectra revealed the clustering of specimens from the same species on the same branch for each *Culex* species for legs (Figure 1c) and thoraxes (Figure 2c). The proximity and low distance of branches between *Cx.* (*Mel.*) *idottus* and *Cx.* (*Mel.*) *rabanicolus* for the thorax body part confirmed the proximity of their MS spectra. However, the absence of species interlacing emphasized the reproducibility and specificity of the protein profiles for both body parts. Although a clustering of *Culex* species from the same subgenus was obtained on both MSP dendrograms, the separation was clearer for MS spectra from legs than from thoraxes. Interestingly, the ordination of the species on the MSP dendrogram was similar to those obtained in the phylogenetic tree from the *COI* gene sequences of the 34 *Culex* specimens (Supplementary File S2).

### 3.3. Leg and Thoraxe Biomarkers Distinguishing Culex Species

To identify discriminatory MS peaks among the 13 *Culex* species for legs and thoraxes, MS spectra from the 169 specimens were analyzed using the GA tool from ClinProTools 2.2 software. As the accuracy of identification is directly linked to the intensity of MS spectra, the analysis was carried out on the most intense mass peaks from legs and thoraxes per mosquito species. After verification of the peak report in the average spectrum, the selection of the top five mass peak lists per species led to a total of 29 and 26 MS peaks for legs and thoraxes, respectively (Supplementary Files S4 and S5). The inclusion of these MS peak lists in the GA model displayed respective recognition capability (RC) and cross-validation (CV) values of 93.2% and 91.4% for legs and 94.5% and 86.5% for thoraxes. When the top ten mass peak lists per species was selected, a total of 37 and 39 MS peaks were found for legs and thoraxes, respectively. The application of the GA model on the top ten mass peak lists did not improve RC and CV values for legs, 92.8% and 91.4%, respectively, whereas, for thoraxes, the CV reached 90.1% and the RC remained at 93.7%. These results underlined that the top five mass peak list per species appeared as the main MS peaks to discriminate these *Culex* species for both body parts. The comparison of the top five mass peak list between legs and thoraxes indicated that three mass-to-charge ratios were similar (at about 5206, 5405 and 6426 m/z), confirming the high specificity of MS spectra per body part (Supplementary Files S4 and S5).

### 3.4. Creation of the MS Reference Spectra Database and Validation Steps

**Figure 3:**
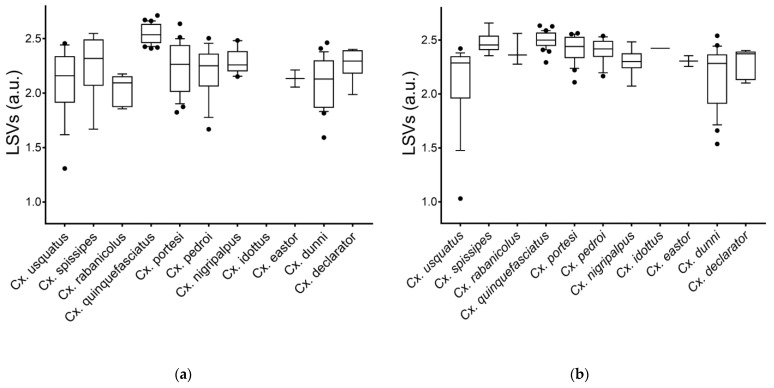
LSVs from query against homemade MS reference database. MS spectra of legs (**a**) and thoraxes (**b**) from *Culex* mosquitoes are presented. Dashed line represents the threshold value (LSV ≥ 1.8) for relevant identification. a.u., arbitrary units; LSV, log score value.

MS spectra from 12 and 13 *Culex* species for legs (*n* = 24) and thoraxes (*n* = 34), respectively, including one to four specimens per species, were selected for database creation (Table 1). MS spectra from the remaining specimens (legs, *n* = 145; thoraxes, *n* = 135) were queried against the DB. The LSVs ranged from 1.31 to 2.71 for legs (Figure 3a) and from 1.03 to 2.66 for thoraxes (Figure 3b). For a reliable identification, an LSV higher than the 1.8 threshold is required [42,56]. In these conditions, correct identification (LSVs > 1.8) could be considered for 95.9% (*n* = 139/145) of legs MS spectra and 94.8% (*n* = 128/135) for thoraxes.

Among these MS spectra reaching the LSVs threshold, 100% of the identification results were concordant with the molecular results. Interestingly, if paired samples per specimen were considered (*n* = 133), solely two specimens of *Cx.* (*Cux.*) *usquatus* failed to obtain an LSV higher than 1.8 for at least one body part (Supplementary File S6). If the LSV cut-off is increased at 2.0 to improve identification confidence, the rate of the specimens that reached this threshold reduces to 78.2% (*n* = 104/133) for legs and 90.2% (*n* = 120/133) for thoraxes. However, 91.0% (*n* = 121/133) of paired samples per specimen achieved this higher threshold in at least one body part.

## 4. Discussion

MALDI-TOF MS is a method routinely used in microbiological diagnostic laboratories for the identification of bacteria and archaea [59], but the relevance of the technique remains largely underestimated for the identification of multicellular organisms. The success of the MALDI-TOF MS for arthropod identification in this last decade highlights this emerging tool as a relevant alternative for mosquito species identification [43,60,61,62]. As many mosquito species from the *Culex* genus have been proven to be vectors of pathogenic agents of human and veterinary importance [63,64,65], accurate classification of the specimens is primordial. The present work demonstrated that MALDI-TOF MS can be used as an alternative to current methods for *Culex* female identification at the adult stage, using legs and thorax.

Prior to MS submission, a relevant classification of the *Culex* mosquitoes per species was compulsory. Firstly, the morphological identification was performed allowing us to classify mosquitoes from only five distinct species, two from the *Culex* subgenus (*Cx.* (*Cux.*) *quinquefasciatus* and *Cx.* (*Cux.*) *usquatus*) and three from the *Melanoconion* subgenus (*Cx.* (*Mel.*) *portesi, Cx.* (*Mel.*) *pedroi* and *Cx.* (*Mel.*) *spissipes*). The accuracy of this morphological classification was confirmed by molecular DNA barcoding of the *COI* gene. The morphological method failed to identify, at the species level, 30% (*n* = 61) of *Culex* mosquitoes. The limitation of the classification of mosquitoes by the morphological approach was also reported for other genera [36,66,67]. For instance, in the *Anopheles* genus, members of the Gambiae Complex and Funestus Group could not be distinguished uniquely by examining external features of their anatomy [68,69]. Moreover, the skill and regular training of entomological experts is also another factor playing a role in the capacity of correct specimen identification [70]. The genus *Culex* is recognized as a highly diverse group of mosquitoes for which delimitation and identification of species is particularly difficult. Complementary methods are therefore required for improving species identification.

DNA barcoding using the mitochondrial *COI* gene is widely used for species identification in molecular taxonomy [71]. A first *COI* barcoding database, available in the BOLD system, established based on morphological classification of male *Culex* specimens of FG [3] was used to validate morphological identification and to classify the unidentified females of this study. The *COI* gene has been recorded as an effective and accessible DNA barcode that provides high performance in delimiting species within the subgenus *Melanoconion* of the *Culex* species, a subgenus displaying the highest species diversity in tropical regions [66]. However, other studies also applied the *COI* barcode fragment for identification of species within the subgenus *Culex* and concluded that this barcode do not contain enough information to distinguish species within this subgenus [36]. Based on *COI* sequences from Brazil and Argentina, Laurito et al. [36] highlighted that *Cx.* (*Cux.*) *declarator* cannot be differentiated from *Cx.* (*Cux.*) *bidens* (Dyar, 1922) and *Cx.* (*Cux.*) *tatoi* (Casal & García, 1971). However, these last two species have never been detected in FG [3], and only *Cx.* (*Cux.*) *declarator* was considered here. Similarly, *COI* sequences of *Cx.* (*Cux.*) *usquatus* can be confused with *Cx.* (*Cux.*) *camposi* (Dyar, 1925), *Cx.* (*Cux.*) *coronator*, *Cx.* (*Cux.*) *maxi* Dyar, 1928 and *Cx.* (*Cux.*) *surinamensis* Dyar, 1918 [36]. In FG, only *Cx.* (*Cux.*) *coronator*, *Cx.* (*Cux.*) *surinamensis* and *Cx.* (*Cux.*) *usquatus* have already been reported. Nevertheless, a recent review of *Culex* species from FG revealed that specimens identified as *Cx.* (*Cux.*) *coronator* in the historical literature were closer to the lectotype of *Cx.* (*Cux.*) *usquatus* described from Suriname [3]. *Culex* (*Cux.*) *usquatus* is morphologically distinguishable from *Cx.* (*Cux*.) *surinamensis*, thus confirming that *COI* sequences classified as *Cx.* (*Cux.*) *usquatus* could be attributed to this species in this study.

To improve the identification, molecular tools often develop new algorithms of analysis or add other molecular markers. For *Anopheles* species, *COI* is not enough to discriminate species of the Gambiae Complex. The addition of ITS2 analysis increases greatly the potential for identification of these species [72]. Indeed, the lack of understanding of *COI* sequences analysis highlights the need to explore complementary or innovative tools.

The proteomic MALDI-TOF MS approach is increasingly used for rapid arthropod identification [42,43,60,61,73]. Indeed, to improve the intra-species reproducibility of MS spectra and to share reference protein profiles, protocols were standardized [44,56]. In order to develop an accessible mosquito spectra database of *Culex* species, the present study employed a MALDI-TOF MS double spectra biotyping strategy. Here, for each specimen tested and for DB creation, legs and thorax from the same specimen were independently submitted to MS analysis whenever possible. Specific MS spectra from the 13 *Culex* species per body part were obtained. The cluster analysis on the MSP dendrogram confirmed this species specificity. In addition, similar ordination of the specimens for both body parts with the *COI* phylogenetic tree revealed a relationship between molecular taxonomic classification and MS spectra profiles. However, as MS spectra from closely related species were grouped in the same branch of the MS dendrogram, a mismatching could occur. To prevent misidentification, the legs and thorax were independently queried against the DB. This double query allowed us to corroborate the identification of each specimen using both body parts, enhancing the identification confidence [55]. In this study, 100% of the samples were correctly identified at the species level on both body parts, and the rate of relevant identification (LSVs > 1.8) was about 95% for each one. Interestingly, a higher reproducibility of MS spectra per species was obtained for thoraxes compared to legs. The lower reproducibility of legs could be attributed to the number of legs available for each specimen. Effectively, legs are breakable and during the catching or storing period some could be lost, which could induce heterogeneity of MS profile intensities among specimens from the same species [74]. In the cases of all the legs being lost, the specimen could be always identified using the thorax compartment. Then, the thorax appears as the more appropriate compartment for mosquito identification, followed by legs, accordingly to previous work [44]. Furthermore, these two body parts do not prevent screening for viruses, parasites or source of blood feeding, which can be researched in the head or abdomen by molecular [75,76] or MALDI-TOF MS methods [77,78]. These complementary data are necessary in the framework of surveillance programs.

The quality of MS spectra can be altered by various factors such as sample homogenization, quantity of mix buffer, engorgement status, storage conditions or even geographical origin of the collection [42,55,74]. The storage is also critical for field-caught mosquitoes. The best method for long storage of arthropods for MALDI-TOF MS analysis is freezing or to maintain them at RT with silica gel [79] when immediate freezing is not possible. In this study, all the specimens were field-derived, dried and stored frozen at −20 °C in tubes or plates for one to three years. The elevate intra-species reproducibility and inter-species specificity of MS spectra for both body parts suggested that specimens were appropriately preserved. Among the 169 mosquitoes tested, only two *Cx. usquatus* failed, on both body parts, to reach the relevant threshold value (LSV > 1.8). These low LSVs could be attributed to improper storage of the mosquitoes. It is possible that protein degradation occurred in these specimens, leading to lower quality of MS spectra, as reported in previous studies [58,80,81].

It is noteworthy that all the *Culex* species selected in this study were correctly classified using MALDI-TOF MS profiling, with concordant results between thorax and legs, and in agreement with molecular identification. Three species from the *Melanoconion* subgenus, *Cx.* (*Mel.*) *idottus*, *Cx.* (*Mel.*) *phlogistus* and *Cx.* (*Mel.*) *rabanicolus*, presenting isomorphic traits among females and which could not be accurately distinguished morphologically, were unambiguously identified by this proteomic tool.

The present spectral database, created using thoraxes and legs from females of 13 *Culex* species from FG, represents the first attempt to create a MALDI-TOF MS database for the identification of neotropical *Culex* species. However, the database encompasses only 12.5% (*n* = 13/104) of the total number of *Culex* species known in FG and solely two out of the eight subgenera from the *Culex* genus of the mosquitoes actually inventoried in the territory [3]. The widening of this MALDI-TOF MS spectra reference DB with missing *Culex* species becomes compulsory for the application of this tool in the monitoring of *Culex* genus vectors in FG. An upgrading of this DB including other mosquito species occurring in FG (*n* = 242) will improve its usage and may be helpful to discriminate cryptic or closely related species. MS spectra for the identification of eight distinct *Anopheles* species from FG, among which four are malaria vectors, are already available [54].

Several works reported the efficiency of the MALDI-TOF MS for the identification of mosquitoes at immature stages [82,83] and demonstrated its relevance in management of Culicidae larval habitats [84]. As larvae can be confusing to distinguish morphologically, it could be interesting to assess MALDI-TOF MS for identification of immature stages. MALDI-TOF MS appears then to be highly promising for discriminating mosquito fauna [41,56,81].

The present work demonstrated that MALDI-TOF MS could be an alternative to current methods for the identification of *Culex* females using dissected legs and thorax. The double body part protein signature of each specimen improved the identification quality. Raw MS spectra from legs and thoraxes of these 13 *Culex* species included in the DB are freely available (Supplementary File S1). The sharing of reference MS spectra is essential in the framework of the creation of an international DB. Except for the expensive cost of the MALDI-TOF MS instrument, this approach is highly competitive economically compared to current molecular methods. It does not require particular skills and could be used for “live” monitoring of vectors due to its short time of handling and obtaining results. This approach appears to be suitable for the identification of mosquitoes from the *Culex* genus, for which morphological and molecular methods remain time-consuming and with a potential risk of misidentification. In the near future, the widening of this reference MS spectra DB with specimens from others *Culex* species, at both adult and immature stages, could improve knowledge of these species for adapted surveillance and control measures to reduce the risks of pathogen transmission not exclusively to French Guiana.

## Figures and Tables

**Table 1:** Overview of *Culex* mosquito origins and identification by *COI* molecular typing.

	Collection					
Species	Site	Year (Month)	Number of Specimens	BOLD# Accession Number (Number of Associated Sequences)	*COI* Gene Sequence Coverage (%)/Identity (%)	Number of Specimens Included in the Reference MS DB per Body Part (Thoraxes/Legs) §
*Cx.* (*Mel.*) *adamesi*	Mac.	2018 (Nov.)	1	FGMOS2220-20 (1)	99%/97%	1/1
*Cx.* (*Cux.*) *declarator*	Mac.	2018 (Nov.), 2019 (Sep.)	7	FGMOS2272-20 (7)	100%/99–100%	2/1
*Cx.* (*Mel.*) *dunni*	Mac.	2018 (Nov.), 2019 (Sep.)	30	FGMOS2748-20 (20); FGMOS2750-20 (10)	100%/98–100%	4/3
*Cx.* (*Mel.*) *eastor*	Mac.	2018 (Nov.), 2019 (Sep.)	3	FGMOS2695-20 (1); FGMOS2743-20 (1); FGMOS2752-20 (1)	100%/99–100%	1/1
*Cx.* (*Mel.*) *idottus*	Mac.	2018 (Nov.)	2	FGMOS3098-23 (2)	100%/100%	1/0
*Cx.* (*Cux.*) *nigripalpus*	Mac.	2018 (Nov.), 2019 (Sep.)	12	FGMOS225-16 (12)	100%/100%	3/2
*Cx.* (*Mel.*) *pedroi*	Mac.	2018 (Nov.), 2019 (Sep.)	15	FGMOS2700-20 (12); FGMOS2758-20 (3)	100%/100%	4/2
*Cx.* (*Mel.*) *phlogistus*	Mac.	2019 (Sep.)	1	FGMOS1542-20 (1)	100%/99%	1/1
*Cx.* (*Mel.*) *portesi*	Mac.	2018 (Nov., Dec.)	28	FGMOS2416-20 (28)	100%/100%	4/1
*Cx.* (*Cux.*) *quinquefasciatus*	Cay.	2019 (Sep.), 2020 (May)	34	FGMOS2275-20 (34)	100%/100%	4/4
*Cx.* (*Mel.*) *rabanicolus*	Mac.	2018 (Nov.)	5	FGMOS2744-20 (5)	100%/100%	2/2
*Cx.* (*Mel.*) *spissipes*	Mac.	2018 (Nov.), 2019 (Sep.)	9	FGMOS2701-20 (9)	100%/97–99%	3/2
*Cx*. (*Cux.*) *usquatus*	Mac., Rem.	2018 (Nov.), 2019 (Nov.)	22	FGMOS046-16 (9); FGMOS049-16 (1); FGMOS2284-20 (12)	100%/99–100%	4/4
Total			169			34/24

#BOLD: Barcode of Life Data Systems; *COI*: cytochrome oxidase subunit 1; §: number of specimens used to create the reference MS database per body part; *Cx*.: *Culex*; *Cux*.: *Culex*; *Mel*.: *Melanoconion*; Mac.: Macouria; Cay.: Cayenne; Rem.: Remire-Montjoly.

## Data Availability

The datasets of MS reference spectra added to the MS DB in the current study are freely available and downloadable from the Supplementary File S1.

## Supplementary Materials

The following supporting information can be downloaded at: https://www.mdpi.com/article/10.3390/tropicalmed8030168/s1, Supplementary File S1: Raw MS spectra from legs and thoraxes of mosquitoes added to the MS reference database; Supplementary File S2: Phylogenetic tree of COI gene from *Culex* genus; Supplementary File S3: Representative legs MS spectra of *Culex idottus* specimens; Supplementary File S4: Top five mass peak list per mosquito species using legs; Supplementary File S5: Top five mass peak list per mosquito species using thoraxes; Supplementary File S6: Comparison of paired body parts LSVs from MS spectra of Culex species.
