# Supplementary material for: Identification of Neotropical Culex Mosquitoes by MALDI-TOF MS Profiling
Source: Trop Med Infect Dis. 2023 Mar 13;8(3):168. doi: 10.3390/tropicalmed8030168 (PMC10055718; doi:10.3390/tropicalmed8030168)
Supplement: Supplementary file 1 [file tropicalmed-08-00168-s001.zip › Additional_file S4.pdf]

Additional file S4. Top-five mass peak list per mosquito species using legs.

MS peak number§	m/z (Da)	Average peak intensity (a.u.)*										
		Cx. declarator	*Cx. dunni*	*Cx. eastor*	*Cx. idottus*	*Cx. nigripalpus*	*Cx. pedroi*	*Cx. portesi*	*Cx. quinquefasciatus*	*Cx. rabanicolus*	*Cx. spissipes*	*Cx. usquatus*
1	2952.56	3.28	**9.56**	8.07	5.35	4.54	7.61	5.13	2.55	9.58	3.54	2.35
2	3108.38	**9.41**	**10.95**	**12.12**	**7.56**	7.31	**9.63**	**10.94**	4.51	8.82	**10.24**	4.23
3	3125.26	4.48	**9.35**	6.14	6.45	4.89	**13.4**	5.49	4.7	**12.39**	6.72	3.38
4	3136.01	6.4	5.43	5.93	7.16	**18.87**	5.62	4.97	2.65	7.37	4.75	7.36
5	3443.81	6.09	8.27	7.58	5.32	11.06	**14.45**	6.87	2.87	8.62	3.55	2.77
6	4073.42	3.47	7.8	10.18	**9.47**	4.52	3.56	3.11	3.31	5.47	5.6	2.66
7	4099.27	4.21	5.36	4.64	4.12	9.42	5.74	3.91	**12.16**	5.9	2.44	3.68
8	4283.69	8.89	8.95	**10.63**	**7.28**	8.47	7.32	5.69	8.04	5.75	5.19	**12.81**
9	4532.03	4.98	8.1	**12.9**	**10.5**	5.66	3.07	3.76	5.64	**11.9**	5.12	5.38
10	4563.79	3.56	**29.32**	**17.56**	2.76	5.65	**10.57**	4.91	3.29	**14.82**	5.08	3.72
11	4590.63	3.29	5.77	4.41	2.26	2.45	6.1	**31.4**	1.76	5.35	**15.83**	2.41
12	4606.79	2.65	4.22	4	2.4	1.66	**31.48**	8.17	1.49	2.31	5.21	1.61
13	4686.19	**16.14**	1.97	1.87	2.24	**16.86**	1.79	1.68	**34.52**	1.61	2.01	2.64
14	4695	**17.73**	2.52	2.16	2.45	**25.77**	1.42	1.93	**8.32**	1.88	1.63	2.94
15	4721.48	**30.21**	2.8	2.55	4.24	**17.64**	2.12	1.83	5.13	3	2.22	**10.36**
16	4819.94	5.24	**14.05**	**10.78**	2.33	5.06	4.76	6.17	**10.43**	2.65	2.06	7.43
17	4933.81	5.23	4.22	5.21	2.09	3.96	3.94	4.19	1.8	2.67	**17.72**	3.87
18	4974.12	1.85	1.87	2.09	**11.15**	3.34	1.62	1.69	4.06	**15.83**	2.14	6.51
19	5206.88#	**12.73**	2.15	3.09	2.15	**14**	3.42	7.35	7.33	3.95	2.25	4.97
20	5405.69#	2.16	8.2	9.42	6.26	1.8	1.59	**9.17**	1.28	6.16	1.56	4.35
21	5495.25	3.32	4.92	5.88	3.55	2.62	5.86	**11.26**	3.3	4.89	6.45	3.12
22	5616.4	2.28	1.46	1.44	2.42	1.28	1.95	**14.95**	2.47	2.11	2.24	1.53
23	6427.33#	3.13	1.53	1.64	1.39	1.7	4.74	2.76	**12.08**	1.1	0.83	2.35
24	6811.21	7.1	1.13	1.19	1.09	6.52	3.92	3.66	6.01	0.8	1.02	**9.51**
25	7447.78	5.27	1.5	0.96	1.45	7.02	0.92	1.15	7.7	1.15	1.56	**19.05**
26	7856.69	4.96	5.73	6.41	2.9	4.78	6.33	5.05	5.58	4.78	**10.19**	2.63
27	8232.11	5.33	1.39	1.31	0.92	1.53	0.77	1.52	7.38	0.66	2.07	**8.78**

28	8658.23	2.95	0.91	0.99	1.86	1.73	0.87	1.43	3.57	**10.6**	1.2	1.26
29	8801.36	0.62	0.67	0.69	1.1	0.5	0.58	1.48	0.68	1.65	**8.73**	0.69

§List of MS peaks used to distinct *Culex* species based on the Genetic Algorithm model analysis of ClinProTools. *The top-five mass peaks per *Culex* species are indicated in bold. [#]MS peaks for which mass-to-charge ratio (m/z) were similar with thorax top-five MS peak list (see Additional file S4). Da: Daltons; m/z: mass to charge; a.u.: arbitrary unit.
