# Supplementary material for: Identification of Neotropical Culex Mosquitoes by MALDI-TOF MS Profiling
Source: Trop Med Infect Dis. 2023 Mar 13;8(3):168. doi: 10.3390/tropicalmed8030168 (PMC10055718; doi:10.3390/tropicalmed8030168)
Supplement: Supplementary file 1 [file tropicalmed-08-00168-s001.zip › Additional_file S5.pdf]

Additional file S5. Top-five mass peak list per mosquito species using thoraxes.

		Average peak intensity (a.u.)*										
**MS peak number§**	**m/z (Da)**	***Cx. declarator***	***Cx. dunni***	***Cx. eastor***	***Cx. idottus***	***Cx. nigripalpus***	***Cx. pedroi***	***Cx. portesi***	***Cx. quinquefasciatus***	***Cx. rabanicolus***	***Cx. spissipes***	***Cx. usquatus***
1	3148.3	8.72	2.86	**18**	**19.9**	8.66	4.16	3	13.74	**14.63**	**18.9**	12.27
2	3196.38	2.33	3.97	2.15	1.65	3	2.71	**15.75**	3.77	2.31	2.01	2.38
3	4081.85	3.78	9.38	6.8	11.28	**20.72**	11.36	9.14	**24.57**	10	7.63	**18.51**
4	4307.06	4.7	2.45	3.13	1.82	13.33	11.71	**16.98**	2.12	1.76	1.59	16.15
5	4324.51	**18.26**	1.87	2.28	2.54	7.36	4.01	5.59	19.77	4.31	2.2	7.5
6	4351.68	6.23	14.31	14.71	**17.19**	3.85	4.44	4.3	5.7	12.42	13.92	4.68
7	5127.88	4.19	**17.67**	3.61	2.83	**4.28**	4.21	2.18	8.94	2.85	2.72	1.76
8	5206.16#	**34.32**	2.08	1.32	1.68	32.02	**14.22**	15.05	**44.38**	2.19	1.99	**36.63**
9	5335.07	3.98	1.36	2.73	2.61	4.22	**15.1**	3.09	4.36	3.19	1.86	3.58
10	5405.68#	1.27	**21.72**	**31.97**	**25.05**	1.38	1.48	**18.89**	1.5	**22.66**	2.28	1.59
11	5450.25	1.2	3.28	2.9	3.65	1.28	2	2.63	1.23	3.35	**18.01**	1.35
12	6085.39	15.15	2.68	17.39	14.94	12.87	5.52	3.29	**20.55**	12.6	6.92	14.79
13	6124.41	4.13	12.1	3.96	4.06	3.76	**16.12**	10.48	5.69	3.62	14.62	4.59
14	6299.52	16.22	5.9	**31.93**	**33.92**	**15.31**	4.78	3.93	**23.54**	**25.93**	**37.42**	**21.75**
15	6357.55	2.6	**33.13**	3.27	3.82	2.57	1.76	3.65	3.09	3.63	4.49	2.98
16	6396.18	1.94	4.79	2.25	1.81	1.76	3.3	**24.36**	2.24	2.12	2.96	2.63
17	6426.43#	1.83	3.87	4.1	1.74	1.38	**17.89**	6.28	4.66	2.4	2.05	3.59
18	8056.24	5.28	3.86	3.04	3	2.08	2.59	4.81	1.59	2.24	**20.2**	2.37
19	8076.8	**19.73**	2.76	3.85	3.96	2.1	2.22	10.32	2.07	2.83	6.03	2.27
20	8163.7	5.1	12.45	10.25	15.04	**35.07**	**17.35**	8.8	**31.34**	**14.09**	8.32	**25.88**
21	8204.74	**20.82**	4.45	2.92	3.57	5.47	3.11	11.62	5.59	3.57	3.78	4.83
22	8613.83	5.84	1.55	2.03	1.75	**17.8**	11.22	**19.69**	4.07	1.64	1.37	**18.26**
23	8646.95	**23.55**	2.19	2.28	3.04	8.02	4.06	5.96	20.49	5.24	3.87	7.13
24	8703.28	6.58	**20.43**	**19.7**	**21.16**	4.16	2.69	4.19	5.73	**16.01**	**21.94**	4.32
25	8945.17	12.43	3	**19.02**	16.5	11.96	8.31	7.25	10.85	13.15	2.5	5.46
26	9016.11	3.2	**17.47**	5.59	4.22	6.07	4.23	14.69	4.95	3.9	17.34	3.05

§List of MS peaks used to distinct *Culex* species based on the Genetic Algorithm model analysis of ClinProTools. *The top-five mass peaks per *Culex* species are indicated in bold. [#]MS peaks for which mass-to-charge ratio (m/z) were similar with legs top-five MS peak list (see Additional file S3). Da: Daltons; m/z: mass to charge; a.u.: arbitrary unit.
