# Supplementary material for: Identification of Neotropical Culex Mosquitoes by MALDI-TOF MS Profiling
Source: Trop Med Infect Dis. 2023 Mar 13;8(3):168. doi: 10.3390/tropicalmed8030168 (PMC10055718; doi:10.3390/tropicalmed8030168)
Supplement: Supplementary file 1 [file tropicalmed-08-00168-s001.zip › Additional_file S6.pptx]

## Slide 1
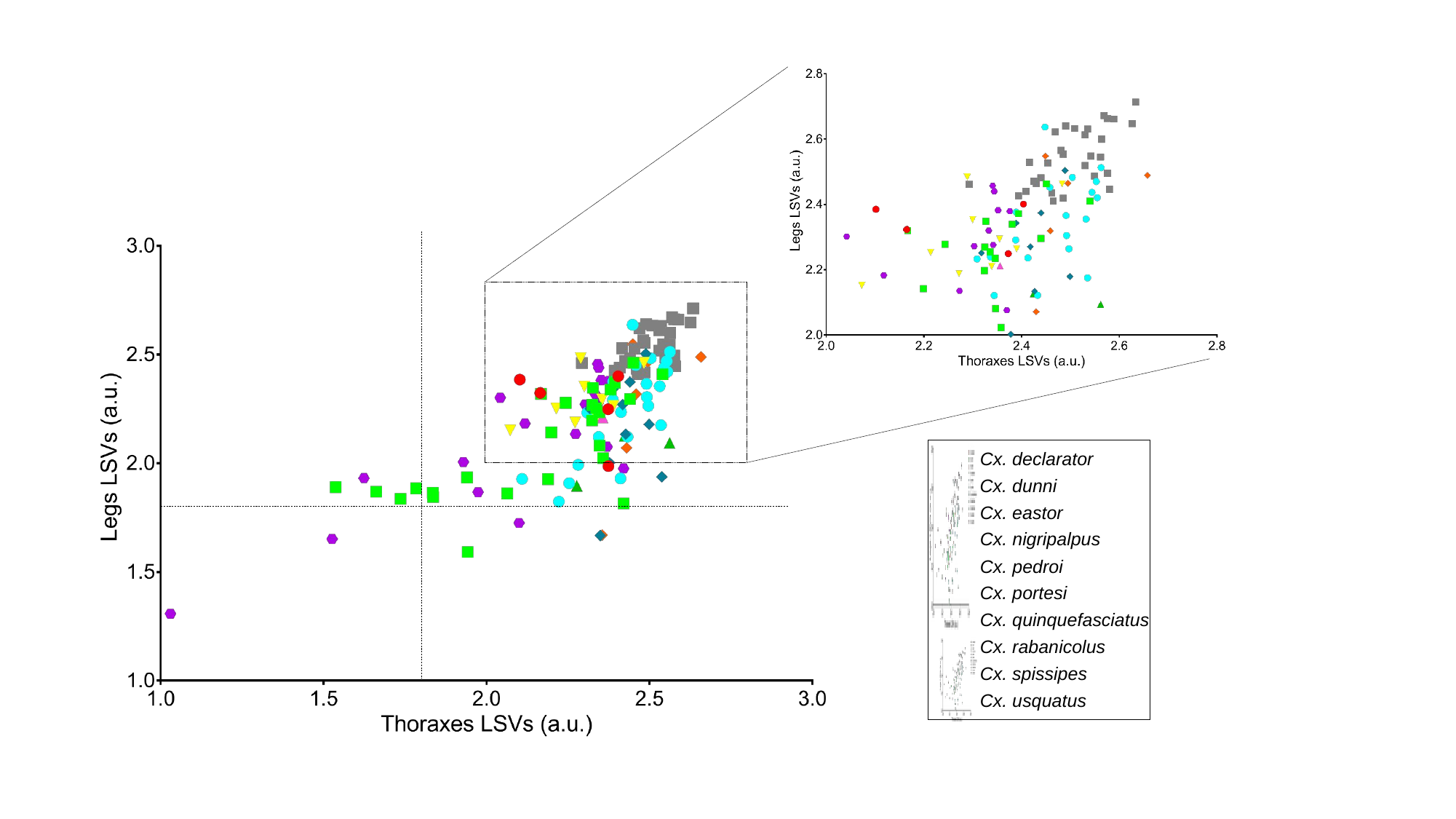

Cx. declarator
Cx. dunni
Cx. eastor
Cx. nigripalpus
Cx. pedroi
Cx. portesi
Cx. quinquefasciatus
Cx. rabanicolus
Cx. spissipes
Cx. usquatus
