# Supplementary material for: Identification of Neotropical Culex Mosquitoes by MALDI-TOF MS Profiling
Source: Trop Med Infect Dis. 2023 Mar 13;8(3):168. doi: 10.3390/tropicalmed8030168 (PMC10055718; doi:10.3390/tropicalmed8030168)
Supplement: Supplementary file 1 [file tropicalmed-08-00168-s001.zip › Additional_files_legend_Costa_et_al.pdf]

Additional files:

Additional file S1. Raw MS spectra from legs and thoraxes of mosquitoes added to the MS reference database. MS spectra were obtained using a MicroFlex LT MALDI-TOF Mass Spectrometer (Bruker Daltonics) and details are presented in the excel file.

Additional file S2. Phylogenetic tree of COI gene from *Culex* genus. Unrooted Maximum Likelihood tree of the 36 *Culex* specimens for which Legs and thorax MS spectra were included in the MS database and with, for each species, an associated reference sequence in purple.

Additional file S3. Representative legs MS spectra of *Culex idottus* specimens. Excluded MS spectra of legs from the two *Cx. idottus* specimens due to the low intensity and inter-sample heterogeneity of MS profiles. a.u., arbitrary units; Cx., *Culex*; m/z, mass-to-charge ratio.

Additional file S4. Top five mass peak list per mosquito species using legs.

Additional file S5. Top five mass peak list per mosquito species using thoraxes.

Additional file S6. Comparison of paired body parts LSVs from MS spectra of *Culex* species. Dashed lines represent the threshold values (LSV ≥ 1.8), for relevant identification. An enlargement of LSVs upper than 2 from MS spectra obtaining for both legs and thoraxes is presented. a.u., arbitrary units; LSV, log score value.
