# Supplementary figures and images for: Identification of Neotropical Culex Mosquitoes by MALDI-TOF MS Profiling
Source: Trop Med Infect Dis. 2023 Mar 13;8(3):168. doi: 10.3390/tropicalmed8030168 (PMC10055718; doi:10.3390/tropicalmed8030168)

## Slide 1
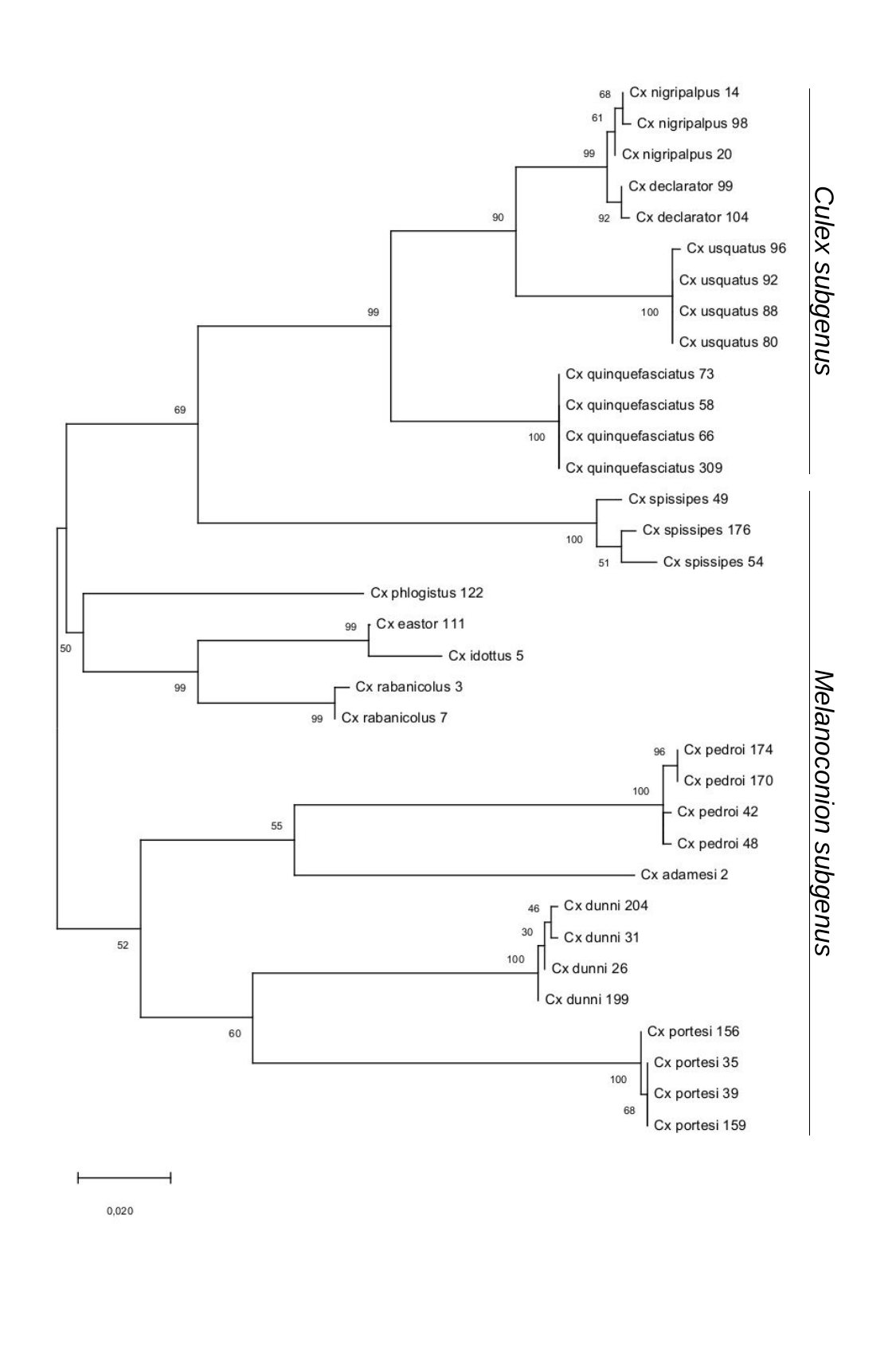

Culex subgenus
Melanoconion subgenus

Supplement: Supplementary file 1 [file tropicalmed-08-00168-s001.zip › Additional_file S2.pptx]

## Slide 1
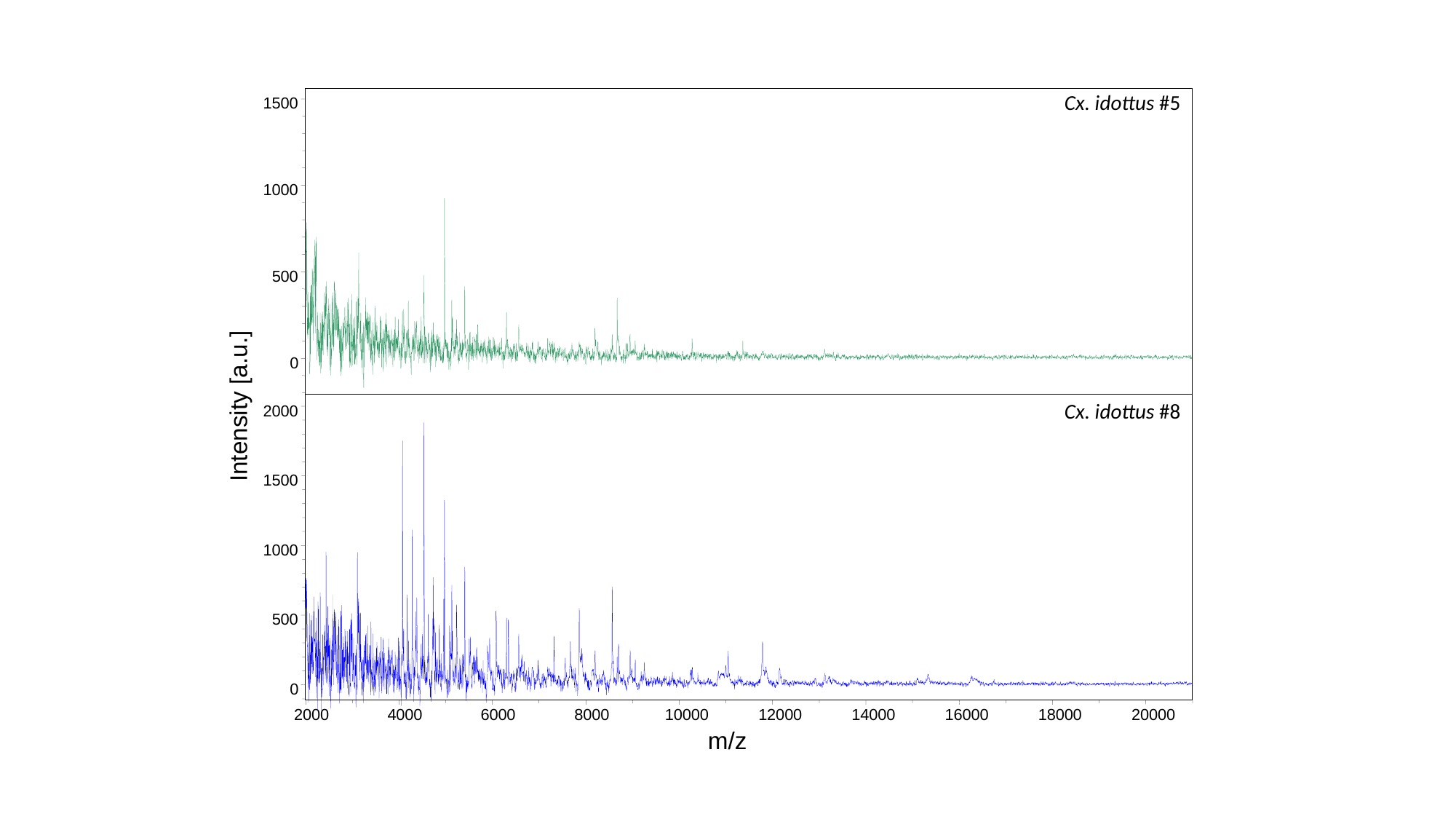

Cx. idottus #5
1500
1000
500
0
2000
1500
1000
500
0
2000
4000
6000
8000
10000
12000
14000
16000
18000
20000
Intensity [a.u.]
Cx. idottus #8
m/z

Supplement: Supplementary file 1 [file tropicalmed-08-00168-s001.zip › Additional_file S3.pptx]
